# Field-Induced Crystalline-to-Amorphous Phase Transformation on the Si Nano-Apex and the Achieving of Highly Reliable Si Nano-Cathodes

**DOI:** 10.1038/srep10631

**Published:** 2015-05-21

**Authors:** Yifeng Huang, Zexiang Deng, Weiliang Wang, Chaolun Liang, Juncong She, Shaozhi Deng, Ningsheng Xu

**Affiliations:** 1 State Key Laboratory of Optoelectronic Materials and Technologies, Guangdong Province Key Laboratory of Display Material and Technology, School of Physics and Engineering; 2 Instrumental Analysis and Research Centre, Sun Yat-sen University, Guangzhou 510275, People’s Republic of China; 3 Sun Yat-sen University-Carnegie Mellon University (SYSU-CMU) Shunde International Joint Research Institute, Shunde 528300, People’s Republic of China

## Abstract

Nano-scale vacuum channel transistors possess merits of higher cutoff frequency and greater gain power as compared with the conventional solid-state transistors. The improvement in cathode reliability is one of the major challenges to obtain high performance vacuum channel transistors. We report the experimental findings and the physical insight into the field induced crystalline-to-amorphous phase transformation on the surface of the Si nano-cathode. The crystalline Si tip apex deformed to amorphous structure at a low macroscopic field (0.6~1.65 V/nm) with an ultra-low emission current (1~10 pA). First-principle calculation suggests that the strong electrostatic force exerting on the electrons in the surface lattices would take the account for the field-induced atomic migration that result in an amorphization. The arsenic-dopant in the Si surface lattice would increase the inner stress as well as the electron density, leading to a lower amorphization field. Highly reliable Si nano-cathodes were obtained by employing diamond like carbon coating to enhance the electron emission and thus decrease the surface charge accumulation. The findings are crucial for developing highly reliable Si-based nano-scale vacuum channel transistors and have the significance for future Si nano-electronic devices with narrow separation.

Nano-scale vacuum channel transistors, relying on the ballistic electron transport in vacuum, are favorable for a variety of potential applications, *i.e.*, sensors, ultra-high speed transistors and THz amplifiers^1^,^2^,^3^,^4^,^5^. The vacuum channel transistors are regarded as the promising alternative to the traditional solid-state field effect transistors, sparking a fresh hint for high frequency, low energy loss, and temperature and radiation immunity^6^,^7^. Already, intensive studies have been performed on the fabrication and characterization of the vacuum channel transistors, which were realized by employing nano-scale field electron emission cathodes^2^,^3^,^4^,^5^,^6^,^7^,^8^,^9^. Device performance including the emission current-voltage characteristic, emission current stability and frequency response were investigated. Although significant progresses have been achieved, the device integration and improvement on lifetime, uniformity and reliability are still open issues.

Specifically, the nano-scale vacuum channel transistor has a narrow vacuum channel in a length of mean free path for electrons in air (between 10 and 100 nm^1^). A high electric field is presented across the channel. Several phenomena pertaining to the effects of high field on semiconductor^10^ and metal surface^11^,^12^ have been studied theoretically and experimentally. P. G. Muzykov and co-workers^11^ found that the electric field evaporation of metal ions from the electrode surface would be able to instigate the breakdown of the vacuum gap. Y. Tomoda *et al.*^12^ have demonstrated that the separation of the nickel nano-gap would decrease by field-emission-induced electromigration. However, there is little understanding on the dramatic changes in the lattice of a semiconductor cathode subjected to an ultra-high electric field, especially insofar as the intriguing observation of their crystalline-to-amorphous phase transformation.

With regard to the well-understood electronic property and the possibility of integration with various monolithic circuits, Si nanostructures have been widely introduced to the fabrication of nano-scale vacuum channel transistors^2^,^3^,^6^. Very less attention has been paid on the atomic-scale material-related mechanisms to the reliability of the vacuum nano-channel, particularly the cathode reliability, under a high electric field. In this work, we report the field induced surface crystalline-to-amorphous phase transformation of the Si nano-emitters. The physical mechanism was proposed by considering the strong electrostatic force on the electrons that accumulated in the high dopant density surface, which is based on the First-Principle calculations with density functional theory (DFT). Furthermore, systematical field emission measurements were performed on the DLC coated individual tips, which confirms reducing the charge accumulation on the apex surface of the Si tip is a commendable solution to “reinforce” the surface crystalline structures and achieve the highly reliable Si nano-cahtodes. The findings are crucial for developing highly reliable Si-based nano-scale vacuum channel transistors and may have the significance for future Si nano-electronic device with narrow separation.

## Results and Discussion

**Figure 1:**
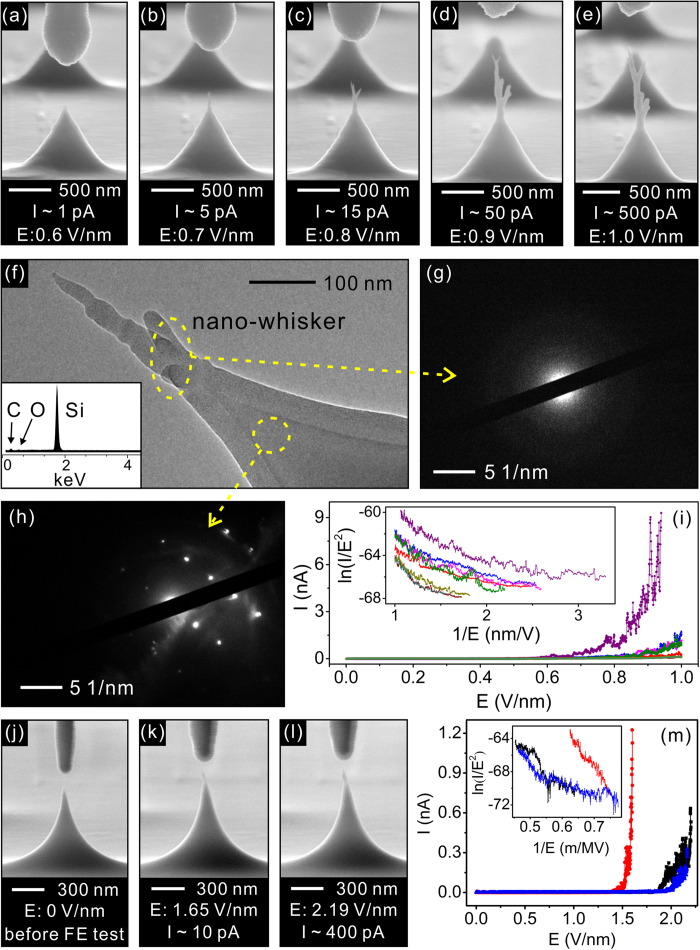
**(a)~(e)** The typical SEM images illustrating the deformed individual Si tip emitter in sequence following the increase of the applied field. The cathode-to-anode separation is 500 nm. **(f)** The typical TEM image of a Si nano-apex with a whisker on top; the inset is the corresponding EDX spectra of the apex. **(g)** The typical SAED image of the nano-whisker. **(h)** The typical SAED image of the bulk of the Si tip. **(i)** The typical field emission I-E characteristics of the individual tips in the 500 nm cathode-to-anode separation tests. The inset is the corresponding F-N plots. **(j)~(l)** The typical SEM images showing the deformed Si tip in sequence following the increase of the applied field. The cathode-to-anode separation is 100 nm. **(m)** The typical field emission I-E characteristics and the corresponding F-N plots of the 3 tested tips in the 100 nm cathode-to-anode separation tests.

Figures 1(a)-(e) show the typical scanning electron microscopy (SEM) images of the deformed individual Si nano-tip apex in sequence following the increase of the applied electric field. The test were performed with a cathode-to-anode separation of 500 nm. Initially, the Si tips were uniform in profile with 1.0 μm in height and the tip apexes were 2~5 nm in radius (see Supplementary Fig. S1(a) online). It was found that the apex started to deform at a low macroscopic electric field of ~0.6 V/nm with an extremely low emission current of ~1 pA. The apex changed its tip shape into a nano-whisker-like structure. At the early stage of the test, the nano-whisker grew in length (Fig. 1(b)). Afterward, the appearance of the nano-whisker changed into a tree-like-structure. The whisker length would still grow following the increase of the applied field (Fig. 1(c)-(e)), accompanying with a higher emission current. The deformation of the tip apex is a typical phenomenon that observed from all the 8 tested nano-tips. In Fig. 1(f), the typical transmission electron microscope (TEM) and selected area electron diffraction (SAED) images of the apex demonstrate that the whisker is in amorphous state. Compared with the original Si nano-apex (the thickness of native oxide layer is ~1 nm, see Supplementary Fig. S1(a) online), the thickness of the surface amorphous layer on the sidewall significantly increases (*i.e.*, ~40 nm, see Supplementary Fig. S2(a) and (b) online). No clear boundary can be found to suggest where the amorphous region starts. The results imply that the amorphization happens on the whole tip surface. Although the tip cannot fully avoid the oxidation in the vacuum level of 10^−4^ Pa, as indicated in the energy-dispersive X-ray spectra (EDX; the inset in Fig. 1(f)), very less oxygen content was detected in the nano-whisker or the sidewall. The atomic ratio of C, O, and Si in the nano-whisker is typically 1:2:73. This evidence proves that the nano-whisker structure at the Si nano-apex is mainly composed by amorphous Si, but not SiO_2_. Figure 1(h) is the typical SAED image of the bulk of the Si tip, showing that the inner core is still in well crystalline. Both the TEM and SAED results indicate that the crystalline-to-amorphous phase transformation happened locally at the Si tip surface. In addition, the EDX analysis (see Supplementary Fig. S3 online) indicates that there are traces of Si on the anode tip apex (6.77% and 14.71% in weight ratio and atomic ratio, respectively). The result suggests that some Si atoms would deposit onto the anode. In Fig. 1(i), it is clearly indicated that the field emission current *vs* the electric field (I-E) characteristics of the tips are in poor uniformity. Typically, breakdown events tend to happen if the electric field exceeds 0.9-1.0 V/nm with an emission current of several nano-ampere. We further changed the cathode-to-anode separation to see if the deformation still happens. In a 50 nm cathode-to-anode separation, the deformation of the cathode (grown up whisker) would result in a contact of the cathode and anode and cause a breakdown. When changing the cathode-to-anode separation to 100 nm, clear deformation was observed from all the 3 tested tips (Fig. 1(j)-(m)). The results suggest that the deformation mechanism is still applied for the measurement with a narrower cathode-to-anode separation.

According to the earlier literatures^13^,^14^, ion bombardment is the main concern for running the field emission devices in a relatively lower vacuum level (*i.e.*, 10^−4^ Pa). The ion bombardment usually results in erosion of the tip emitter and an eventually breakdown of the cathode^14^. However, the field-induced-growth of the nano-whisker (even up to a length of ~1.0 μm) (Fig. 1(e)) and a very smooth sidewall (Fig. 1(f)) was observed in our experiment. This is different from the usual ion bombardment phenomenon. In addition, the ion bombardment phenomenon is usually accompanied with an electro-static discharge^15^. However, in our observations, the apex started to deform at a low macroscopic electric field of ~0.6 V/nm with an extremely low emission current of ~1 pA. No upheaval in emission current was recorded. Thus, we hold the idea that the vacuum condition would make less significant effect on our findings.

Moreover, the deformation of the tip apex may cause either by the field induced evaporation^16^,^17^,^18^ or the electromigration^11^,^19^,^20^,^21^ of the specific atoms. In our experiments, the direction of the applied electric field was opposite to that in the field evaporation model^17^,^18^. Thus, we turned to the possibility related to the electromigration. In the electromigration model^20^,^21^, the basic process involves motion of atoms from the cathode to anode, imparted by the momentum of “electron-wind”. Electromigration is the result of the combination of thermal and electrical effects on mass transport. Large current is needed to raise the temperature high enough to promote the atomic mobility. However, in our observations, the deformation of the Si tip happened at an ultra-low emission current (~1 pA). The numerical simulation turned out that the temperature of the nano-apex was about 27.9 °C, strongly suggesting that less Joule-heating effect might be taken into account for the observations. It is not coincident with the “electron-wind” theory of the electromigration model.

**Figure 2:**
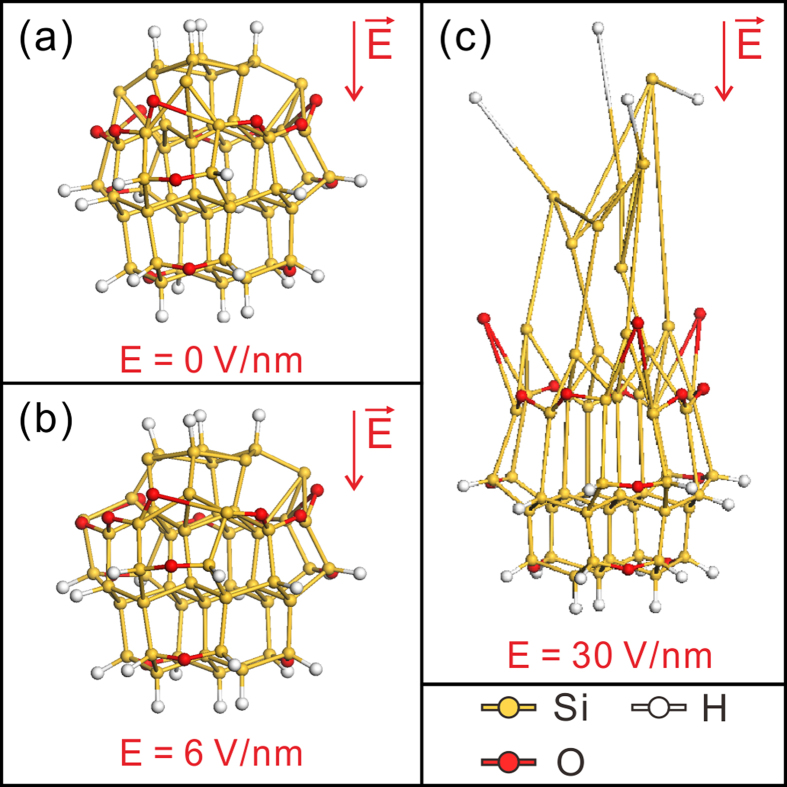
**(a)** The spheroidal atomic model with 54 Si atoms terminated with hydrogen (-H) and oxygen (-O) atoms. **(b)** The Si_54_H_22_O_15_ at 6 V/nm, showing an elongated Si-Si bond length. **(c)** The deformed Si_54_H_22_O_15_ cluster under the critical field of 30 V/nm.

In order to understand this unusual deformation phenomenon, numerical simulations based on First-Principle calculations were performed with the density functional theory implemented in DMol^3^. In the simulation, we define a spheroidal atomic model of 54 Si atoms terminated with hydrogen (-H) and oxygen (-O) atoms (Fig. 2(a)). It is reasonable to present H and O in the model, since it has been widely accepted that there is a native oxide layer and the hydrogen adsorption on the surface of crystalline Si^22^,^23^. In the model, the H and O saturation can efficiently prevent surface reconstruction of the Si_54_H_22_O_15_ cluster, forming a stable cluster. It was found that the deformation of the Si_54_H_22_O_15_ cluster is induced by the electrostatic force exerting on the atoms, which has strong dependence on its “Mulliken charges”. The “Mulliken charges” is defined to be the distribution of the electron cloud around the specific atoms^24^. High density charge is accumulated in the top atoms of the Si_54_H_22_O_15_ cluster under the action of the electric field. It results in a stronger electrostatic force exerting on these accumulated charges, causing the redistribution of the electron cloud around the Si-Si covalent bond. As indicated in Fig. 2(b), the Si-Si bond length is elongated with the increasing electric field (also see the movie file in the Supplementary Information online). We define the critical field (*E*_*Crit*_) for the deformation to be the electric field needed for inducing the top atoms to depart from their nearest neighbor one by a distance twice to that of the original separation (Fig. 2(c)). The simulated *E*_*Crit*_ of the Si_54_H_22_O_15_ cluster is 30 V/nm and the structure of the cluster becomes disordered under such a high electric field.

Though the simulations were based on a Si_54_H_22_O_15_ cluster, the results give a clue that the local charge accumulation in the Si tip surface has significant effect on the amorphization of the tip apex. In the present work, ultra-low emission current was obtained when the nano-apex started to deform. It was suggested that less of the electron could emit into the vacuum while high density electron was accumulated in the tip surface. That is because of the relatively high surface electron affinity of Si (*i.e.*, 4.1 eV). A strong electrostatic force was exerted on the surface lattices, causing the redistribution of the electron cloud around the Si-Si covalent bonds. The bone length of these Si-Si bonds in the surface lattices was elongated. It would lead to weaker Si-Si bonds and result in a crystalline-to-amorphous phase transformation. The accumulated electrons were located at the field penetrated surface layer. Therefore, the phase transformation was confined to the surface (Fig. 1(f)). In addition, the strain on the surface lattice would tend to make the amorphization easier. In the crystalline-to-amorphous phase transformation layer, atoms were negatively charged with the reason of the locally high density charge accumulation. The electrons would interact with the positive charged nucleus, and tend to “pull” the nucleus to move along the opposite direction of the electric field. As a result, the atoms in surface crystalline-to-amorphous phase transformation layer of Si nano-apex migrated along the opposite direction of the electric field, causing a formation of the amorphous nano-whisker-like structure on the nano-apex.

The above discussions give a phenomenological model to qualitatively explain the formation of the amorphous nano-whisker-like structure on the Si tip apex during the field emission measurement. It is worth noting that the calculated critical field (30 V/nm) for the deformation is rather high, while the experimental observation happened at a relatively lower field (0.6 V/nm). Thus, more physical insights should be considered. In the typical Si tip fabrication procedure, it is a consensus that thermal oxidation is a crucial step, which can not only sharpen the tip but also obtain well geometrical uniformity^25^,^26^,^27^, Although it has been studied that thermal annealing would induce a dopant re-distribution in the Si nanostructure^28^,^29^, the significance of the properties change in post-fabrication of the Si nano-cathode has not been clearly revealed.

**Figure 3:**
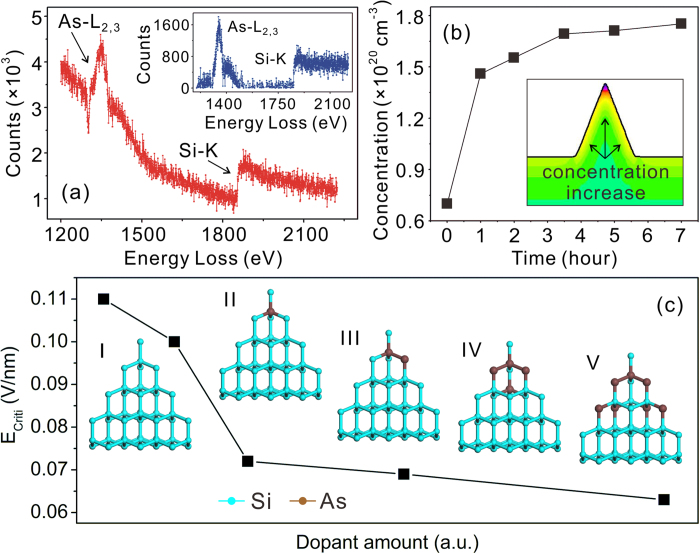
**(a)** The typical EELS spectrum of the tip apex. The inset is the corresponding background removed EELS spectrum. **(b)** The simulated arsenic concentrations at the tip apex with different oxidation durations. The inset shows a typical dopant distribution in a tip with a seven-hour-oxidation. **(c)** A curve showing the *E*_*Crit*_ changing with the amount of the arsenic dopant. The insets are the corresponding structural models of the simulated Si_52_ clusters.

We have employed the electron energy loss spectroscopy (EELS) to investigate the arsenic dopant concentration of the tip apex. The value of arsenic to Si atomic ratio was calculated from the As-L_2, 3_ and Si_K_ edges indicated in the EELS spectra (Fig. 3(a)) by employing a Si volume density of 5 × 10^22^ cm^−3^ 30,31. The typical arsenic to Si atomic ratio is ~1:16, 45 times to that of the primary Si substrate (*i.e.*, ~1:714). We took the thermal sharpening process into account to the surface accumulation of arsenic dopant. Simulations based on Fick’s law were performed with an isosceles trapezoid two-dimensional model. Figure 3(b) shows the typical results of the simulation at 1000 °C. The arsenic concentrations given in the curve are the average value in an area of 20 nm^2^ at the Si tip apex. The arsenic concentration increases nonlinearly from the primary value of 7.00 × 10^19^ cm^−3^ to ~1.75 × 10^20^ cm^−3^ following the increasing oxidation duration. The inset of Fig. 3(b) shows a typical dopant distribution in a tip with a seven-hour-oxidation. The dopant presents an uneven distribution from the bulk to the surface. Both experiments and numerical simulations suggests that the arsenic dopants have been drawn out from the oxide and diffused into the surface of the Si nano-apex during the thermal oxidation, forming an arsenic-rich apex.

The thermal induced arsenic dopant diffusion would result in a high concentration of defect and vacancy in the Si lattice. The high concentration defect and vacancy are crucial for the atomic migration in the lattice^20^. The field-induced amorphization of surface lattices in Si nano-apex involves motion of negatively charged atoms. The atoms (atomic flux *J*_*a*_) in surface crystalline-to-amorphous phase transformation layer migrated along the opposite direction of the electric field while the relatively generated vacancies moved in the field direction. There is still lack of mature theoretical model to describe this field-induced amorphization phenomenon. But we could adopt the traditional atomic migration model on the electromigration of metal surface^20^,^21^,^32^, and make some modifications to explore a solution for theoretically understanding the field-induced amorphization phenomenon in our observations. The *J*_*a*_ can be determined by the following formula
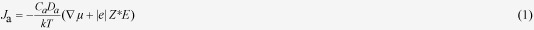
where *C*_*a*_ is the atomic concentration of Si, *D*_*a*_ is the self-diffusion coefficient of Si atom, *|e|* is the absolute value of the elementary charge, *E* stands for the apply electric field. *k* is the Boltzmann constant, and *T* is the temperature in degrees Kelvin. *μ* is the chemical potential function for the atomic diffusion associated with the difference between the atomic (*μ*_*a*_) and vacancy (*μ*_*v*_), *i.e.*, 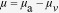. Traditionally, in the electromigration model for atomic migration, the effective charge number Z* is correlate with the free electron flow in metals^20^,^21^,^32^. However, in our observations, free electron flow in Si nano-cathode is insufficient owing to the ultra-low emission current, while the ultra-high electrostatic force acting on the highly density negatively charged atoms in the Si tip surface is the dominant factor to make the atom migration. Therefore, Z* should be defined with new significance as a function of the effective charge number of atoms. The Z* associates with the atomic concentration as well as the electric field in different locations of the tip surface lattices.

In the following, discussions will be presented to clarify the effect brought by each of the quantities in equation (1). Firstly, in the case that the vacancies are in thermal equilibrium *μ*_*v*_ = 0, the chemical potential function *μ* could be determined by the inner stress of the lattice. In equation(1), the chemical potential gradient 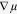 would increase with the growing inner stress of the lattice. The relationship between the inner stress *σ* and the dopant diffusion induced defect and vacancy could be expressed as^32^.
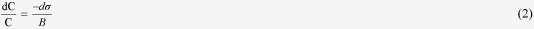
where *C* is the function of the dopant concentration. *B* is the applicable modulus of Si which could be treated as a constant value (*i.e.*, 130 GPa^33^). The arsenic-rich tip surface would contribute higher inner stress in the lattice. Accordingly, there would be a relative higher 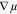 and thus a larger Si atomic flux *J*_*a*_ at a specific applied field. Secondly, due to the fact that the arsenic atom is a donor center to Si, the augment of arsenic atom will increase the “Mulliken charges” for the Si atoms under the action of the electric field. Namely, the effective charge number Z^*^ is increased, inducing a stronger electrostatic force and thus causing a higher Si atomic flux. Finally, owing to the arsenic dopant accumulation, both the local atomic concentration *C*_*a*_ and effective charge number Z^*^ are not constants. Both the *C*_*a*_, and Z^*^ could reach their extreme values at the tip apex. According to the above discussions, all the effects brought by the thermal-induced arsenic dopant accumulation on the Si tip surface can result in a higher atomic flux. This can surely lead to a lower electric field for inducing a crystalline-to-amorphous phase transformation.

Further numerical simulations based on First-Principle calculations have been performed for the verification of the proposed model. As showed in Fig. 3(c), we define a tip-shape cluster of 52 Si atoms (Si_52_) with different amount of arsenic atoms. The simulated E_Crit_ of the primary Si_52_ cluster is 0.110 V/nm, and it is non-linearly decreased from 0.110 V/nm to 0.063 V/nm following the adding of the arsenic atoms. The simulation results fit well to the model. More importantly, the model predicts that in Si nano-electronic devices with nanogaps, especially in the application of high dopant concentration Si nanostructures, such a charge accumulation related deformation would be a crucial problem.

**Figure 4:**
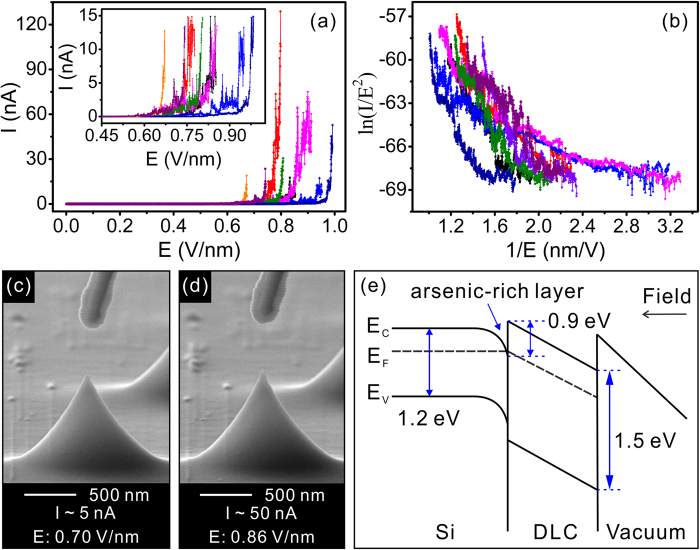
**(a)** The typical field emission I-E curves of 8 DLC coated Si tips. The inset is the detail view of the field emission I-E curves. **(b)** The corresponding F-N plots of the 8 DLC coated Si tips. **(c)~(d)** The typical SEM images the DLC coated Si tip in field emission tests. **(e)** A schematic illustration of the energy band diagram of the Si nano-apex with DLC coating.

According to the above discussion, to employ the lightly doped Si wafer as the substrate, change the type of the dopant (P-type: boron or aluminum), or develop an oxidation-free process are helpful to inhibit the deformation of the Si nano-cathode. However, they may bring disadvantages of high resistance, saturation effect^27^, and non-uniform tip profile with relatively rough surface, respectively. To reduce the charge accumulation can be a good option to restrain the deformation. Here, diamond like carbon (DLC, 3 nm in thickness) has been employed for this purpose. The DLC not only has a low surface affinity for enhancing the field electron emission but also has much stronger C-C bond (85 kcal/mol) than that of the Si-Si ones (54 kcal/mol)^34^. Figure 4(a) show the typical field emission I-E curves and the corresponding Fowler–Nordheim (F-N) plots of 8 DLC coated tips. In the detail view of the field emission I-E curves in the inset of Fig. 4(a), each DLC coated tips can carry a relative lager emission current up to 15.0 nA, showing a clearly superior emission performance than that of uncoated ones. It is worth noting that some of the coated tips can even carry remarkable emission currents (*i.e.*, up to ~128.8 nA). More importantly, no morphology change was observed (Fig. 4(c)) for all the tested tips.

The enhancing filed emission from the DLC coating is mainly contributed by the “quasi-tunneling” of electron through the ultra-thin (3 nm) DLC film^35^. That would reduce both the charge accumulation and the electrostatic force on the surface lattice. Meanwhile, the DLC film with stronger C-C bond can prevent the migration of the surface atoms. Both these effects can “reinforce” the lattice structure of the Si tip. More significantly, the finding of dopant accumulation on apex provides strong evidence to enrich the “quasi-tunneling” model. Based on the finding of the present work, the arsenic dopant concentration is gradually increased from the “bulk” to the Si tip surface. The band gap of Si and DLC are ~1.2 eV and ~1.55 eV, respectively^26^. A Schottky barrier with a finite height of 0.9 eV is formed at the Si/DLC interface (Fig. 4(e)). Owing to the accumulated high-density arsenic dopant, the Si/DLC interface becomes a degenerate semiconductor. It would cause a formation of a metallic-like layer. The Fermi level of Si in the metallic-like layer moves gradually upward close to the bottom of its conduction band. Under this consideration, the potential barrier between the Fermi level of Si (E_F_) and the conduction band of DLC at the Si/DLC interface is close to 0.9 eV, even possibly lower than this barrier. The electron tunneling efficiency from Si to DLC can be significantly enhanced, making an effective emission. Although the dopant accumulation will induce a lower field deformation on the oxidation sharpened Si tips, it brings a much lower Si/DLC interface barrier for enhancing the emission. There is still a lot work to do in the future to clarify how the dopants migrate at high current field emission and the corresponding effect on the emission performance (especially the emission uniformity of the individual tips).

## Conclusions

The crystalline Si tip apex was deformed to amorphous nano-whisker-like structure at a relatively low macroscopic field with an ultra-low emission current. Both experimental and numerical simulation investigations demonstrate that the arsenic dopant atoms tend to accumulate on the Si tip surface during the thermal sharpening, forming an arsenic-rich apex. It will increase the inner stress and result in the charge accumulation in the Si surface lattice. The effect lowers the crystalline-to-amorphous phase transformation field of Si lattice, causing a low-field deformation. DLC thin film was employed to improve the reliability of the Si nano-cathode by taking the advantages of stronger C-C bonds and the enhancing field electron emission. The findings inspire vital insight in the interpretation of the reliability of the Si nano-cathode while can be used as essential approach to obtain highly reliable nano-scale vacuum channel transistors.

## Methods

### Fabrication of Si Nano-Tip Cathod**e**

The mono-crystalline heavily arsenic doped (~10^19^/cm^3^; 0.005 Ω cm) Si substrate was intentionally employed for the fabrication of the nano-tip cathode. The advantage of using heavy doped Si is to utilize the well conductivity, which is beneficial for field electron emission. The Si nano-tip arrays were fabricated following a well-developed top-down procedure^26^,^35^. Electron beam lithography was used to define the dot (1 μm in diameter) pattern of AR-N 7520 resist. The patterns were transfer into SiO_2_ (450 nm in thickness) and followed by a Si anisotropic etch using an inductively coupled plasma etching system (ICP, OXFORD Plasmalab), employing the SF_6_-based etchant. The tips were sharpened by thermal oxidation (1000 °C for 7 hours), followed by oxide removing using hydrofluoric acid. The tip height is typically 1 μm while the separation between the adjacent tips is 6.0 μm. The DLC thin film was deposited on the Si tip surface using a filtered cathodic vacuum arc deposition system^26^,^35^,^36^. The optical band gap of the DLC is ~1.55 eV^26^. The sp^3^ content of DLC thin film is ~80%^36^. The room-temperature conductivity of the films was also measured to be ~4 × 10^−8^ (Ω cm)^−1^ 35.

### Morphology and Structure Investigations

The morphology, structure, and elemental composition of the tips were investigated with a scanning electron microscope (SEM, Zeiss SUPRA 55), transmission electron microscope (HRTEM, JEM-2010HR, 200 kV), and electron energy loss spectroscopy (EELS) on an aberration corrected dedicated scanning transmission electron microscope (STEM, FEI Tecnai F30, 300 kV), respectively. Selective area energy-dispersive X-ray spectroscopy (EDX) equipped in the SEM was employed to analyze the chemical composition of the nano-whisker.

### Field Emission Test

The field electron emission measurements of the individual tips were carried out at room temperature in a SEM system (~1.0 × 10^−4^ Pa) equipped with a piezo-driven nano-motor (Klocke Nanotechnik). A fine tungsten micro-tip was fixed on the nano-motor and be employed as an anode probe for the tests (see Supplementary Fig. S1(b) online). To study the field emission characteristic of the Si nano-cathode, the microprobe was moved to the same focal plane with the tip apex. The separation between the anode probe and Si nano-cathode was set to be 50, 100, and 500 nm. A Keithley 6487 picoammeter was employed to apply bias voltage and recorded the field emission current. The bias voltage was increased manually at a speed of 0.10 V/s. The SEM electron beam was blanked off during the measurement.

### Density Functional Theory (DFT) Calculation

First principle calculations based on DFT were performed to understand the field evaporation of the Si clusters with DMol^3^ code^37^,^38^. The exchange-correlation functional was treated using the Perdew-Wang (PWC) local functional approximation (LDA)^39^. The potential with all-electron was considered in all calculations. The electronic convergence tolerance in the self-consistent field (SCF) calculation was set to 10^−6^ eV. The structures were optimized until the total energy converged below 2.0 × 10^−5^ Ha and the force on atoms converged below 1.0 × 10^−2^ eV/Å. We defined a spheroidal atomic models for 54 Si atoms terminated with hydrogen (-H) and oxygen (-O) in the simulations. The H and O saturation could efficiently prevent surface reconstruction of the Si_54_H_22_O_15_ cluster, making the structure of cluster more stable. In addition, another tip-shape cluster of 52 Si atoms (Si_52_) with different amount of arsenic atoms was further defined to simulate the effect of arsenic dopant concentration on the distorted field of the cluster. In the simulations, the cluster was grounded and faced to the electric field.

### The Simulations of Temperature on Si Nano-apex

The simulations of the temperature on the Si tip apex were carried out by using the commercial software package COMSOL Multiphysics with a 3D axisymmetric model. In the model, the “tip” is with 1.0 μm in height and the tip apex is 5.0 nm in radius. The initial temperature of the tip was set to a uniform value of room temperature (300.15 K). A reasonable value of thermal conductivity (*i.e.*, 50 W/m K) was chosen according to Ref. 40 which is consistent with the size effect of the Si nanostructure in the experiment. Different value of emission current was set from 0.00 pA to 100.00 nA to calculate the temperature at the nano-apex of the “tip”.

### Simulations of the Arsenic Dopant Distribution

The simulations of the arsenic atom diffusion based on Fick’s law were performed by a “SILVACO 11 ATLAS” software. An isosceles trapezoid two-dimension model was established for simulating a “tip”. In the model, the isosceles trapezoid solution domain is 1.0 μm in height, and with 50 nm and 1200 nm top and bottom edges, respectively. The primary concentration of arsenic dopant was set to a uniform value of 7.00 × 10^19^ cm^−3^. The segregation coefficient of arsenic atoms at the Si/SiO_2_ interface is 10^28^. Different value of oxidation time was set from 0 hour to 7 hour to calculate arsenic dopant concentration in the nano-apex of the “tip”.

## Author Contributions

J.C.S. and N.S.X. created the experimental protocols. Y.F.H. carried out the fabrication and characterization of Si nano-cathodes. Z.X.D. and W.L.W. performed the first principle calculations based on DFT. C.L.L. carried out the STEM EELS experiments. Y.F.H., J.C.S. and S.Z.D. performed the experimental and simulation data analysis. The manuscript was written through contributions of all authors.

## Additional Information

**How to cite this article**: Huang, Y. *et al*. Field-Induced Crystalline-to-Amorphous Phase Transformation on the Si Nano-Apex and the Achieving of Highly Reliable Si Nano-Cathodes. *Sci. Rep.* **5**, 10631; doi: 10.1038/srep10631 (2015).

## Supplementary Material

Suplementary Information

Supplementary Movie S2
