# Supplementary material for: Field-Induced Crystalline-to-Amorphous Phase Transformation on the Si Nano-Apex and the Achieving of Highly Reliable Si Nano-Cathodes
Source: Sci Rep. 2015 May 21;5:10631. doi: 10.1038/srep10631 (PMC4440211; doi:10.1038/srep10631)
Supplement: Suplementary Information [file srep10631-s1.pdf]

Supplementary Information

Field-Induced Crystalline-to-Amorphous Phase Transformation on the Si Nano-apex and the Achieving of Highly Reliable Si Nano-cathodes

Yifeng Huang[†], *Zexiang Deng*[†], *Weiliang Wang*[†], *Chaolun Liang*[††], *Juncong She* [*, †,†††], *Shaozhi Deng*[†], *Ningsheng Xu* [*, †]

[†]State Key Laboratory of Optoelectronic Materials and Technologies, Guangdong Province Key Laboratory of Display Material and Technology, School of Physics and Engineering, and [††]Instrumental Analysis and Research Centre, Sun Yat-sen University, Guangzhou 510275, People's Republic of China

[†††]Sun Yat-sen University-Carnegie Mellon University (SYSU-CMU) Shunde International Joint Research Institute, Shunde 528300, People's Republic of China

***Address correspondence to** *shejc@mail.sysu.edu.cn and stsxns@ mail.sysu.edu.cn*

Figure S1(a) shows the typical scanning electron microscope (SEM) image (85 °tilt-view) and transmission electron microscope (TEM) image of the thermal sharpened Si tips array. The tips are uniform in profile, having a typical height of ~1.0 μm. The separation between the adjacent tips is 6 μm. The tip apex is typically 2~5 nm in radius with native oxide (~1 nm in thickness) on the surface. The TEM investigation demonstrates that the Si nano-apex is in well crystallization with lattice direction of [110] (the inset of **Figure S1(a)**). **Figure S1(b)** shows a typical SEM image of a tungsten anode probe facing to an individual tip ready for field emission measurement. The field emission measurements were performed on 8 individual tips. These 8 tips were randomly selected from the diagonal of the array.

In **Figure S2(a) and (b)**, one can clearly see that the entire sidewall of the deformed tip was covered with a smooth amorphous layer. No clear boundary can be found to suggest where the amorphous region starts. The thickness of the amorphous layer is typically ~40 nm. In the typical energy-dispersive X-ray spectra (EDX, **Figure S2(c)**) of this sidewall amorphous layer, very less oxygen content was detected. The atomic ratio of C, O, and Si is typically 1.1:1:38. This evidence proves that the sidewall layer is mainly composed by amorphous Si (but not SiO_2). The results imply that the amorphization happened on the whole tip surface while the atom migration occurred at the tip apex where it has highest local electric field.

Figure S3 shows the typical EDX spectra of the tungsten anode-tip apex, which had experienced the field emission test of the individual Si tip. There are traces of Si been found, i.e., 6.77% and 14.71% in weight ratio and atomic ratio, respectively. The result suggested that some

dissociated Si atoms would deposit onto the anode.

We have also performed numerical simulations about the dopant distribution of the *Phosphorous* doped tip after thermal oxidation and the corresponding field-induced deformation. Simulation based on *Fick's Law* found that, the *Phosphorous* atoms are tending to accumulate on the tip apex during the thermal oxidation (**Figure S4**), showing the same diffusion behavior as that of the *Arsenic* dopant. According to the *First-Principle* calculations with *Density Functional Theory*, the *Phosphorous* doped tip showing relatively higher deformation field, which is mainly due to the stronger Si-P bonding (*i.e.* 363 kJ/mol).

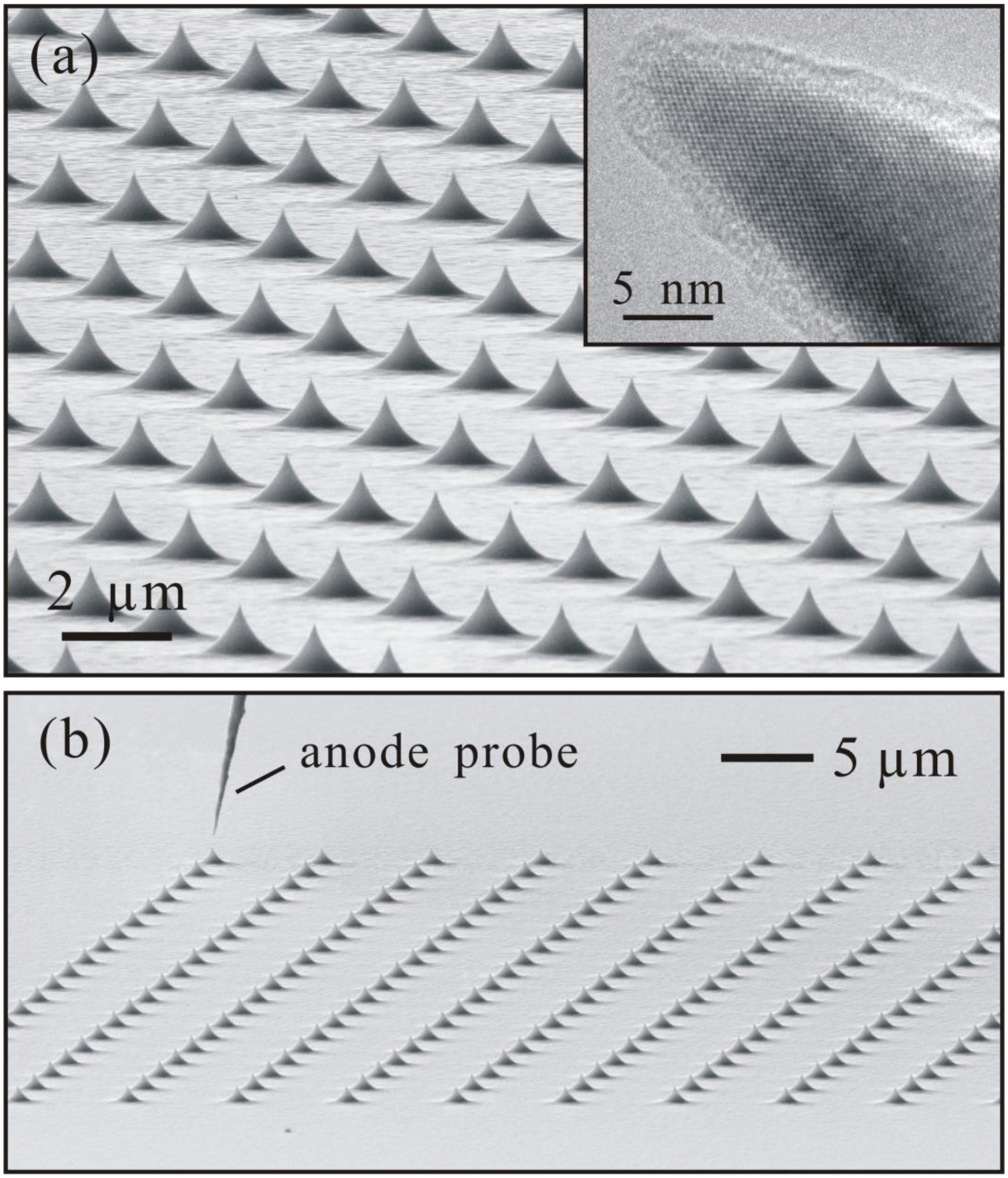

Figure S1. (a) The typical SEM and TEM (the inset) images of the thermal sharpened Si tips. **(b)** A SEM image showing an anode probe facing to an individual tip ready for field emission measurement.

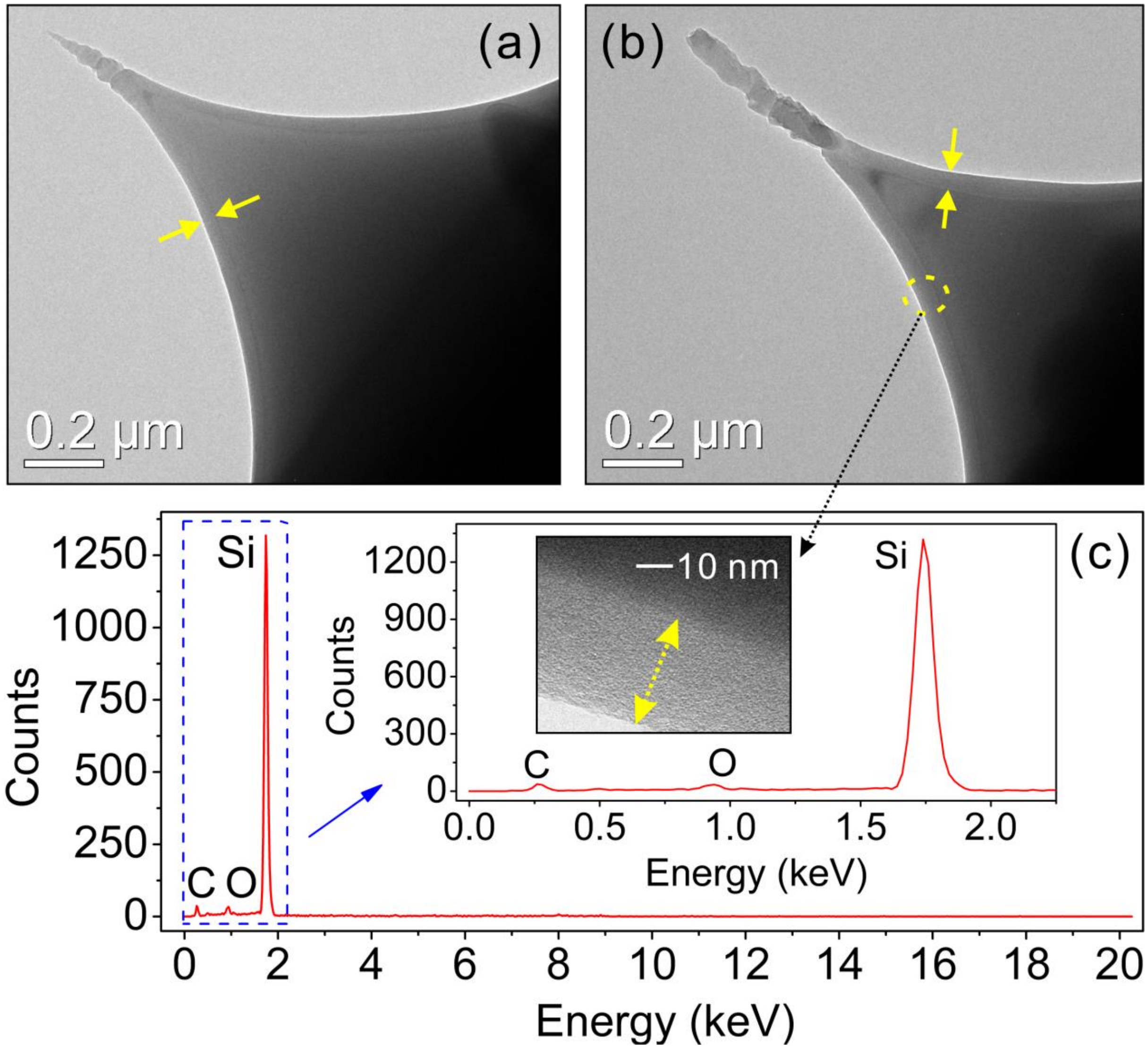

Figure S2. (a) and **(b)** The typical zoom-out TEM images of two different deformed tips. The entire sidewall of the tip was covered with a smooth amorphous layer (arrowed in the figures). **(c)** The typical EDX spectra of the sidewall amorphous layer of the deformed Si tip. The inset is the enlarged TEM image of the amorphous sidewall.

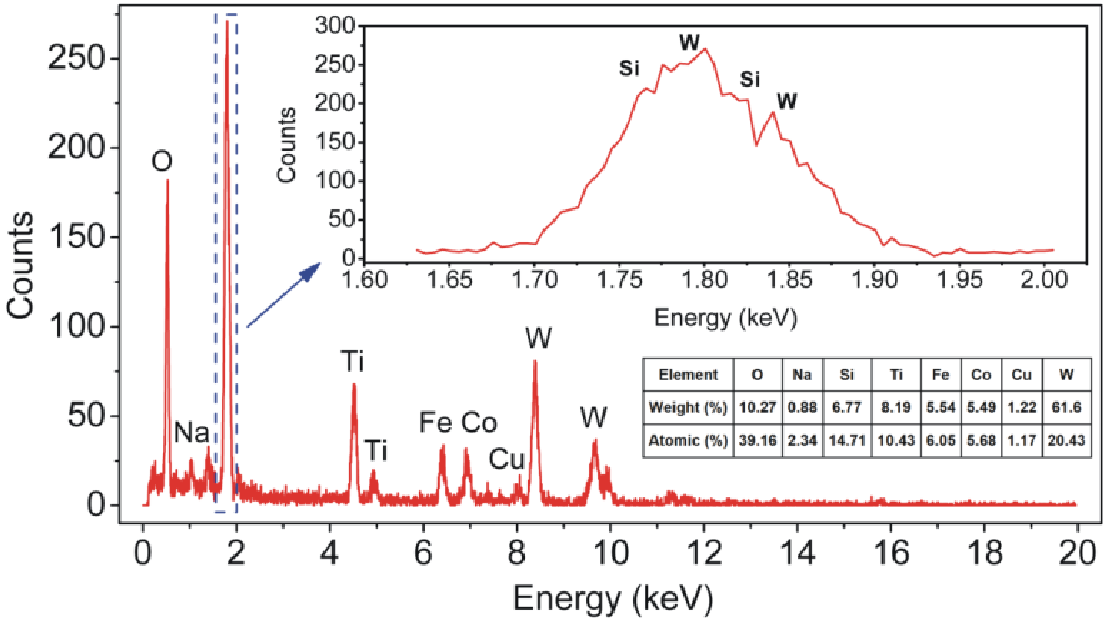

Figure S3. The typical EDX spectra of the tungsten anode-tip that had experienced the field emission tests of the individual Si tip. The Na, Ti, Fe, Co, and Cu signals suggested that tungsten tip is not in good purity.

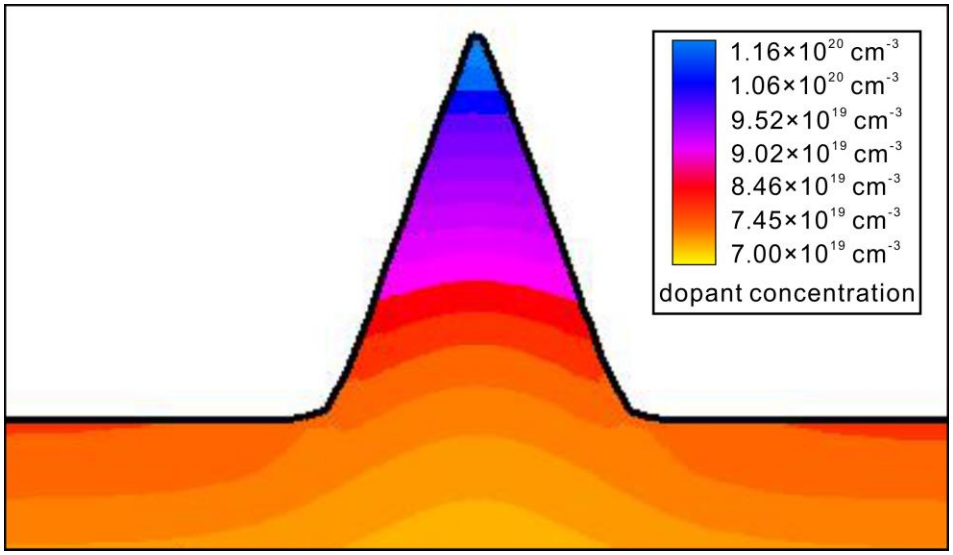

Figure S4 The *Fick's Law* simulation result of the *Phosphorous* dopant distribution in a Si tip with 7-hour-oxidation.

Legend for Video

Video 1. The simulation movie of the deformation on the $Si_{54}H_{22}O_{15}$ cluster.
